# Polyamines Upregulate Cephalosporin C Production and Expression of β-Lactam Biosynthetic Genes in High-Yielding *Acremonium chrysogenum* Strain

**DOI:** 10.3390/molecules26216636

**Published:** 2021-11-02

**Authors:** Alexander A. Zhgun, Mikhail A. Eldarov

**Affiliations:** Group of Fungal Genetic Engineering, Federal Research Center “Fundamentals of Biotechnology” of the Russian Academy of Sciences, 119071 Moscow, Russia

**Keywords:** polyamines, filamentous fungi, biosynthesis of secondary metabolites, *Acremonium chrysogenum*, cephalosporin C

## Abstract

The high-yielding production of pharmaceutically significant secondary metabolites in filamentous fungi is obtained by random mutagenesis; such changes may be associated with shifts in the metabolism of polyamines. We have previously shown that, in the *Acremonium chrysogenum* cephalosporin C high-yielding strain (HY), the content of endogenous polyamines increased by four- to five-fold. Other studies have shown that the addition of exogenous polyamines can increase the production of target secondary metabolites in highly active fungal producers, in particular, increase the biosynthesis of β-lactams in the *Penicillium chrysogenum* Wis 54–1255 strain, an improved producer of penicillin G. In the current study, we demonstrate that the introduction of exogenous polyamines, such as spermidine or 1,3-diaminopropane, to *A. chrysogenum* wild-type (WT) and HY strains, leads to an increase in colony germination and morphological changes in a complete agar medium. The addition of 5 mM polyamines during fermentation increases the production of cephalosporin C in the *A. chrysogenum* HY strain by 15–20% and upregulates genes belonging to the beta-lactam biosynthetic cluster. The data obtained indicate the intersection of the metabolisms of polyamines and beta-lactams in *A. chrysogenum* and are important for the construction of improved producers of secondary metabolites in filamentous fungi.

## 1. Introduction

Filamentous fungi (also called molds or moldy fungi) are taxonomically diverse organisms from phylum *Ascomycota* and *Zygomycota* with filamentous hyphae and the ability to produce airborne spores or conidia. Improved strains of filamentous fungi are one of the most important sources for producing pharmaceutically significant secondary metabolites (SMs), such as antibiotics, statins, and immunosuppressants [1,2,3,4]. These compounds are one of the most commonly prescribed drugs worldwide [5,6]. *Acremonium chrysogenum*-improved strains are the main source for obtaining cephalosporin C (CPC), the parent substance for producing antibiotics of the cephalosporin series of generations 1–5 [7,8,9,10].

*A. chrysogenum* HY (RNCM 408D) strain, the high-yielding CPC producer, was obtained as a result of multi-round random mutagenesis of an *A. chrysogenum* WT (ATCC 11550) [11]. This improved strain typically produces 9–12 g/L of CPC during laboratory fermentation in shake flasks, 200–300 times higher than *A. chrysogenum* WT. The HY strain is one of the most comprehensively characterized among the improved *A. chrysogenum* producers of CPC [2,11]. For this strain, the chromosomal rearrangements compared to WT strain were shown; the number of copies and localization of biosynthetic gene clusters (BGCs) of beta-lactams, the so-called “early” and “late” clusters, were determined [12]. The expression of BGCs genes was measured; “early” and “late” were upregulated 5–300-fold [13]. The *A. chrysogenum* HY was one of the first highly active producers of SM in filamentous fungi, for which a genetic transformation using *Agrobacterium tumefaciens*-mediated transformation (ATMT) was developed [14]. Using this technology, we obtained a series of recombinant strains, expressing CefT (MFS beta-lactam transporter from *A. chrysogenum*) and PMA1 (H^+^-ATPase the plasma membrane from *S. cerevisiae*), which made it possible to study in detail the features of transport and energy consumption of beta-lactam biosynthesis in *A. chrysogenum* [2,15]. The HY strain was also characterized by a shift in several biochemical parameters, particularly the content of five fractions of energy-rich inorganic polyphosphates (polyPs), ATP, and H^+^-ATPase activity in the plasma membrane [16]. Several morphological alterations in cell wall structure [17], in size of filamentous hyphae and conidia formation [14], and in colony size and coloration [15] were also demonstrated.

Recently, for this strain, significant changes in the metabolism of polyamines (PAs) were found, in particular, increased resistance to inhibitors of ornithine decarboxylase (ODC; EC 4.1.1.17), an enzyme of the key stage of PAs biosynthesis [18]. Additionally, the content of major PAs in HY cells during fermentation was increased (the content of spermidine was increased 5.2-fold; spermine was increased 4.5-fold) [18].

There are numerous known functions of PAs in fungi, associated with stress resistance [19], morphogenesis and differentiation of fungal cells [20,21,22], plant pathogenesis [23,24], and many others [25,26]. In the last decade, it was shown that the introduction of exogenous PAs during the fermentation of improved SM-producing filamentous fungi could lead to an additional increase in the production of target SM [27,28]. This effect can be mediated by the work of LaeA, the key global regulator of SMs, which interacts with other components of the velvet complex (VeA, VelB, VelC, and VosA) [29,30,31]. It is so named because four of these proteins, VeA, VelB, VelC, and VosA, have an approximately 150-amino-acid domain called the velvet domain [32]. The velvet domain is a DNA-binding motif that specifically recognizes an 11-nucleotide consensus sequence consisting of two motifs in the promoters of key regulatory genes for fungal development; this domain is also involved in homo- or heterodimer formation [33,34]. These proteins form dimers, heterodimers, and heterotrimeric complexes with other proteins also involved in the regulation of morphogenesis, as well as metabolism and other cellular functions, in response to environmental stimuli, such as light or carbon source [35]. The heterotrimeric velvet complex proteins, LaeA/VeA/VelB, have been most studied in fungi to clarify the relation between light-dependent morphology and secondary metabolism [36]. There are a number of models for the functioning of the velvet complex [34,37]. One of the most important roles of this complex is associated with the global regulation of the secondary metabolism of fungi [38,39,40].

In this regard, the main goal of our work was to find and characterize, at the molecular level, the conditions under which the introduction of exogenous PAs can increase the yield of CPC in an improved *A. chrysogenum* HY strain.

## 2. Results

### 2.1. Effect of PAs on A. chrysogenum Strains on an Agarized Medium

#### 2.1.1. Growth of *A. chrysogenum* WT and HY Strains on an Agarized Complex Medium

**Figure 1:**
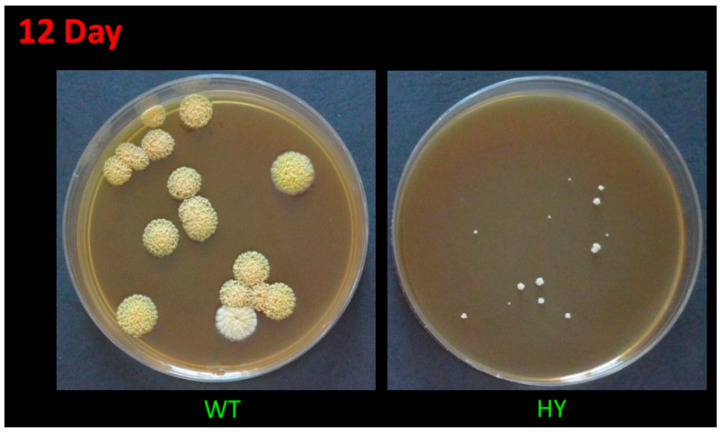
Growth of *A. chrysogenum* wild-type (WT) and high-yielding (HY) strains after 12 days on agarized complex (CPA) medium.

The high-yielding *A. chrysogenum* strain, during the multiround random mutagenesis to improve CPC production, became significantly less viable than the original wild-type strain; in particular, it lost the ability to form conidia [14]. It is also extremely sensitive to freezing at −80 °C (with standard cryoprotectants), lyophilization, and long-term storage at 4 °C on agarized media. *A. chrysogenum* HY cells die after such manipulations. The only stable method of maintaining this strain is sequential subculture on agarized media. However, with this method of storing the culture over time (after 3–4 transfers), low-active clones are released, which significantly reduces the yield of the strain. Such a splitting into low activity and high activity in the production of CPC clones may be due to epigenetic events, since the BGCs’ loci are under the control of factors of global regulation, remodeling chromatin in these regions [41,42]. To maintain a highly active line, it is necessary to conduct periodic monospore sieving on an agarized medium, stimulating the formation of CPC, such as an agarized complex (CPA) medium. This leads to the identification of heterogeneity in the size of the colonies. There are relatively large colonies (low in the production of CPC), medium-sized colonies, about two times smaller (actively producing CPC), as well as small colonies, 5–10 times smaller, with extremely low viability and unsuitable for effective CPC production (Figure 1). At the same time, the largest colonies of *A. chrysogenum* HY are significantly smaller in size (10-fold or more) than colonies in *A. chrysogenum* WT after monospore seeding on CPA medium (Figure 1). Additionally, the wild-type strain does not show heterogeneity in colony size on this medium.

#### 2.1.2. Effect of PAs on the Coloration of *A. chrysogenum* Strains on a CPA Medium

Colonies of *A. chrysogenum* WT have a characteristic yellow-cream color, which develops after 5–10 days, depending on the cultivation conditions on the agar nutrient medium, and due to the biosynthesis of the SM sorbicillin [43]. Single colonies can remain white for up to 12–15 days, but they also acquire a yellow-cream color (Figure 1).

**Figure 2:**
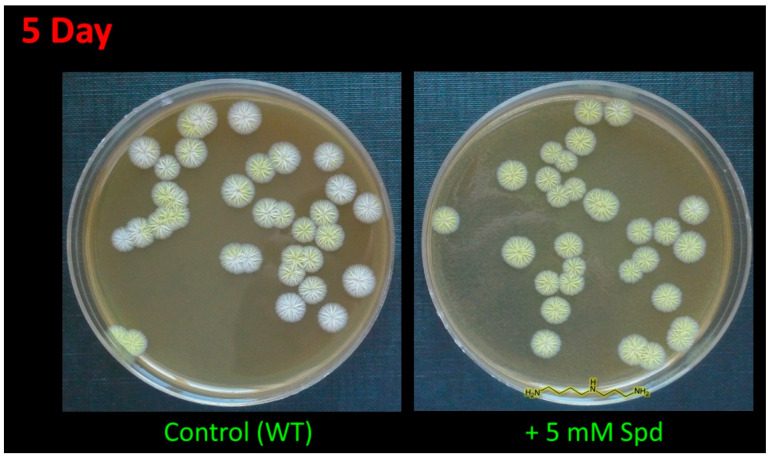
Effect of spermidine (SPD) on pigmentation of *A. chrysogenum* wild type (WT) strain. After 5 days on agarized complex (CPA) medium with the addition of 5 mM Spd or without addition (control).

In the current study, the addition of 5 mM 1,3-diaminopropane (1,3-DAP) or 5 mM spermidine (SPD) led to an earlier appearance of pigment coloration. After 4–5 days of inoculation, colonies grown on the medium supplemented with PAs were completely yellow, while most colonies on the control medium were white or just starting to turn yellow (Figure 2). After 10–15 days, the control strains completely stained yellow and did not differ in color (including its intensity) from cells grown on a medium containing PAs.

The *A. chrysogenum* HY strain lost color pigmentation during the strain improvement (Figure 2) [14]. The addition of PAs to *A. chrysogenum* HY did not change the colony color; they remained white.

#### 2.1.3. Effect of PAs on Germination and Size of *A. chrysogenum* Colonies on CPA Medium

To determine the effect of PAs on the germination and the size of the diameter of *A. chrysogenum* colonies on the CPA medium, 1,3-DAP and SPD were used in the concentration range of 0.1–10 mM.

**Figure 3:**
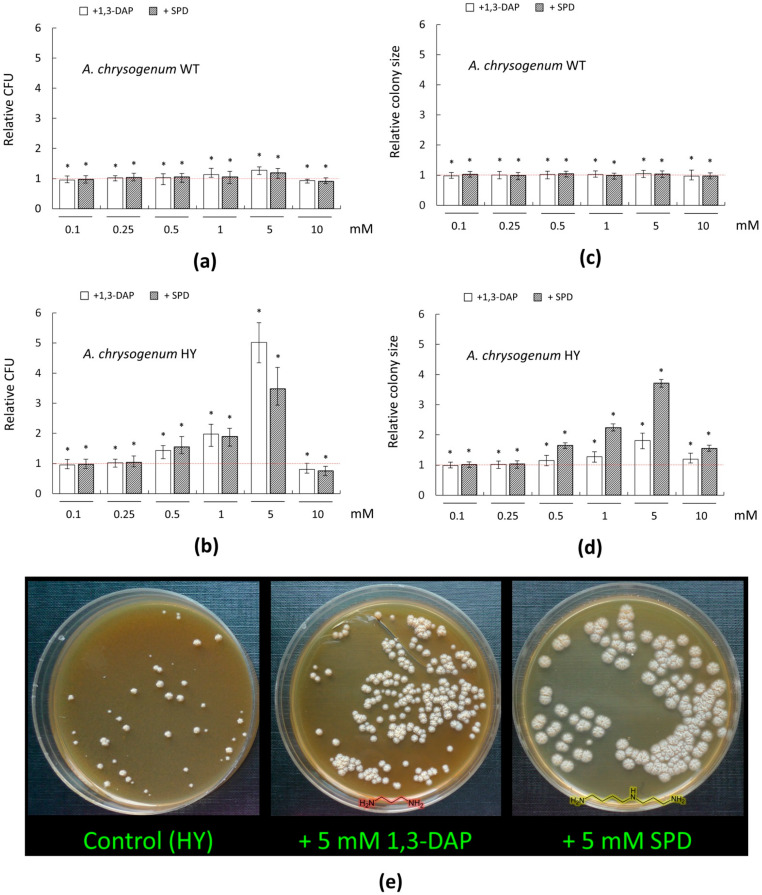
Effect of 1,3-diaminopropane (1,3-DAP) and spermidine (SPD) on the number and diameter size of germinating colonies of *A.* *chrysogenum* strains on agarized complex (CPA) medium: (**a**,**c**) *A. chrysogenum* wild type (WT) strain; (**b**,**d**,**e**) *A. chrysogenum* high-yielding (HY) strain. (**a**,**b**) Relative CFU (colony forming units)—the ratio of the of cell counts after incubation on CPA medium with PAs compared control counts (on CPA medium without any additions). (**c**,**d**) Relative size—the ratio of the average diameter of all colonies after inoculation on CPA with PAs compared to control’s average diameter of colonies (on CPA media without any additions). Dotted lines show the relative CFU level or colony diameter for strains inoculated on CPA without additives. Data are means ± SD, *n* = 3. Statistical significance, * *p* ≤ 0.05, as compared with the control (strain, cultivated on medium without PAs additions). (**e**) 12 days after inoculation of *A. chrysogenum* HY strain on CPA medium with the addition of 5 mM 1,3-DAP or 5 mM Spd, or without addition (control).

For the WT strain, there were no significant changes in the number of germinated colonies and the size of their diameter upon addition of PAs in the range of 0.1–0.5 mM (Figure 3a,c). The addition of 1 mM 1,3-DAP resulted in a small (by 10–15%) increase in the number of CFU/mL; the addition of 1 mM SPD had no significant effect. The addition of 5 mM 1,3-DAP increased CFU/ ml by 25–30%; the addition of 5 mM SPD increased this parameter by 15–20%.

A total of 0.1–5 mM PAs had no significant effect on the colony size of the *A. chrysogenum* WT strain. Small decreases in both growth parameters were observed upon the addition of 10 mM PAs, which may indicate the toxicity of these compounds at such concentrations (Figure 3a,c). Previously, using *Saccharomyces cerevisiae* as a model object, we demonstrated that high concentrations of PAs can be toxic to fungal cells [15]. For this, we studied (i) a series of *S. cerevisiae* strains from the Euroscarf collection, with knockouts for various MDR (multidrug resistance) transporters (BY4741 Δybr043c (ΔQdr3), BY4741 Δypr156c (ΔTpo3), and BY4741 Δyll028w (ΔTpo1) and (ii) based on them recombinant analogs, heterologously expressing *cefT* gene for the MDR transporter from *A. chrysogenum*. The functioning of the CefT transporter in recombinant clones made it possible to more effectively resist the toxic effect caused by the addition of high concentrations of SPD for yeast cells as compared to the control [15]. This shows that PAs in high concentrations are toxic to fungi; the threshold of resistance to toxic concentrations of exogenous PAs can be significantly different for different strains.

We found that adding the studied PAs to the CPA medium could significantly affect germination and colony size of *A. chrysogenum* HY strain (Figure 3b,d,e). The stimulation of germination of colonies and an increase in their size began at concentrations of 0.5 mM and reached the strongest effect at 5 mM.

The addition of 0.5 mM 1,3-DAP or SPD increased the number of germinating colonies by ~1.5-fold; 1 mM of these compounds increased CFU/ ml by ~two-fold. At the 5-mM concentration, PAs had a different degree of stimulating effect. Five mM SPD increased approximately ~3.5-fold CFU/ ml compared to the control; 5 mM 1,3-DAP stimulated this even more—the increase was ~five-fold. The addition of 0.1–0.25 mM PAs had no effect. A 10 mM concentration of 1,3-DAP or SPD was toxic. The number of germinated colonies decreased by 20% with 10 mM 1,3-DAP and even more, by 25%, with the addition of 10 mM SPD.

The PAs addition also led to a change in the phenotype of the colony size of the HY strain on the CPA medium (Figure 3e). The addition of 1,3-DAP in the concentration range of 0.5–5 mM led to a gradual disappearance of heterogeneity in the colony size due to the increase in small and medium colonies to the size of large colonies. The maximum effect of adding 5 mM 1,3-DAP led all colonies of the HY strain to become the same size, corresponding to the size of the large control colonies; as a result, the total average diameter of the colonies increased ~two-fold to the control (Figure 3d). The addition of 0.1–0.25 mM 1,3-DAP did not affect the colony size; 10 mM 1,3-DAP resulted in a sharp decrease in colony size compared to 5 mM 1,3-DAP, which may indicate the toxicity of such a high concentration of this substance.

The addition of SPD caused similar effects of 1,3-DAP: 0.1–0.25 mM concentration had no significant effect, 0.5–5 mM was stimulated with the greatest effect at 5 mM, and 10 mM was toxic to cells. While the general trend was similar, SPD had a significantly more pronounced stimulating effect on increased colony size than 1,3-DAP. Thus, the addition of 5 mM SPD resulted in a significant change in the macromorphology of HY strain colonies. They became twice as large in colony diameter as the largest colonies on the control medium; moreover, they were all the same size (Figure 3d,e).

### 2.2. Submerged Fermentation of A. chrysogenum HY Strain with Exogenous PAs

#### 2.2.1. Optimization of Cultivation *A. chrysogenum* HY Strain with PA to Increase CPC Production

At the first stage, our experiments showed that the addition of exogenous PAs could significantly affect the viability and morphology of *A. chrysogenum* HY on the CPA medium, expressed in an increase in CFU/mL and colony size. In this regard, our further task was to find out whether exposure from exogenous PAs can also lead to an additional increase in the production of CPC.

**Figure 4:**
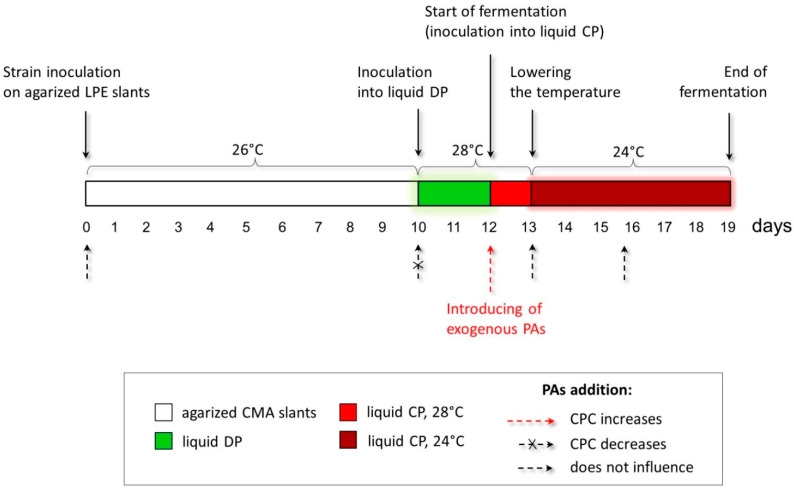
Optimization of the conditions for introducing exogenous polyamines (PAs) to increase cephalosporine C (CPC) production in the *A. chrysogenum* high-yielding (HY) strain. The red dashed arrow shows the optimal time for PAs’ addition to increase CPC production at the inoculation from liquid defined (DP) medium to liquid complex (CP) medium. Small dashed arrows mark the periods of strain cultivation when the addition of PAs does not affect CPC production. The small crossed-out dashed arrow marks the period when the addition of PAs leads to a decrease in CPC production.

The studied *A. chrysogenum* HY strain was improved for the overproduction of CPC during submerged fermentation [11]. It is known that phenotypic effects obtained on agar medium do not always scale [44]. Additionally, fungal strains improved for solid-state fermentation (SSF), and submerged fermentation most efficiently produced the target SM in the environment for which the improvement was made [45]. This is due to both the difference in regulation during SSF and submerged fermentation and many other reasons affecting the biosynthesis of the target SM [46,47]. Therefore, we took the data obtained from the phenotypic responses of *A. chrysogenum* HY to add PAs on the agar medium only as a starting point for optimizing the submerged fermentation. There, we used the concentration of PAs in the range of 0.5–5 mM (since on agar media, concentrations of 0.1–0.25 mM turned out to be ineffective, and the concentration of 10 mM was toxic). In addition to testing different concentrations of PAs, we varied the time of their introduction at the preliminary stages and during the biosynthesis of CPC (Figure 4).

The process of obtaining CPC from *A. chrysogenum* includes several sequential steps associated with obtaining an inoculum on an enriched agar medium, preliminary cultivation on a liquid defined (DP) medium, and growing in a liquid complex (CP) medium with a decrease in temperature after the first 24 h of incubation (Figure 4). We investigated the impact of adding PAs at each stage. In most cases, adding 0.5–5 mM 1,3-DAP or SPD did not result in significant shifts in dry biomass and CPC production.

It turned out that the optimal for increasing production is the introduction of 5 mM 1,3-DAP or 5 mM SPD directly into the CP medium at the time of transfer of the accumulated culture on the DP medium (Figure 4). The increase in the production of CPC is not affected by preliminary cultivation on LPE slants with the addition of PAs. Additionally, there were no differences if the cell culture was carried out through two consecutive passages on a slant agar supplemented with PAs. An increase in CPC production does not occur when exogenous PAs are added to the CP medium after 24 h or at later stages of fermentation. The additions of 5 mM 1,3-DAP or SPD to the liquid DP medium significantly reduced both the yield of CPC and the weight of dry biomass. This concentration of PAs appears to be toxic when added to a low-nutrient DP medium. The addition of 0.5–1 mM PAs had no significant effect on biomass growth and CPC production of the *A. chrysogenum* HY strain.

In this regard, for our further study of the effect of PAs on increasing the production of the target SM in the improved *A. chrysogenum* strain, we used 5 mM 1,3-DAP or 5 mM SPD to be added at 0 h of fermentation, that is, at the time of transfer of the culture from the DP to the CP medium (corresponds to day 13 of work with the strain, according to Figure 4).

#### 2.2.2. Effect of the Addition of 5 mM PAs on CPC Production, Biomass, and Specific CPC Yield during Submerged Fermentation of *A. chrysogenum* HY

**Figure 5:**
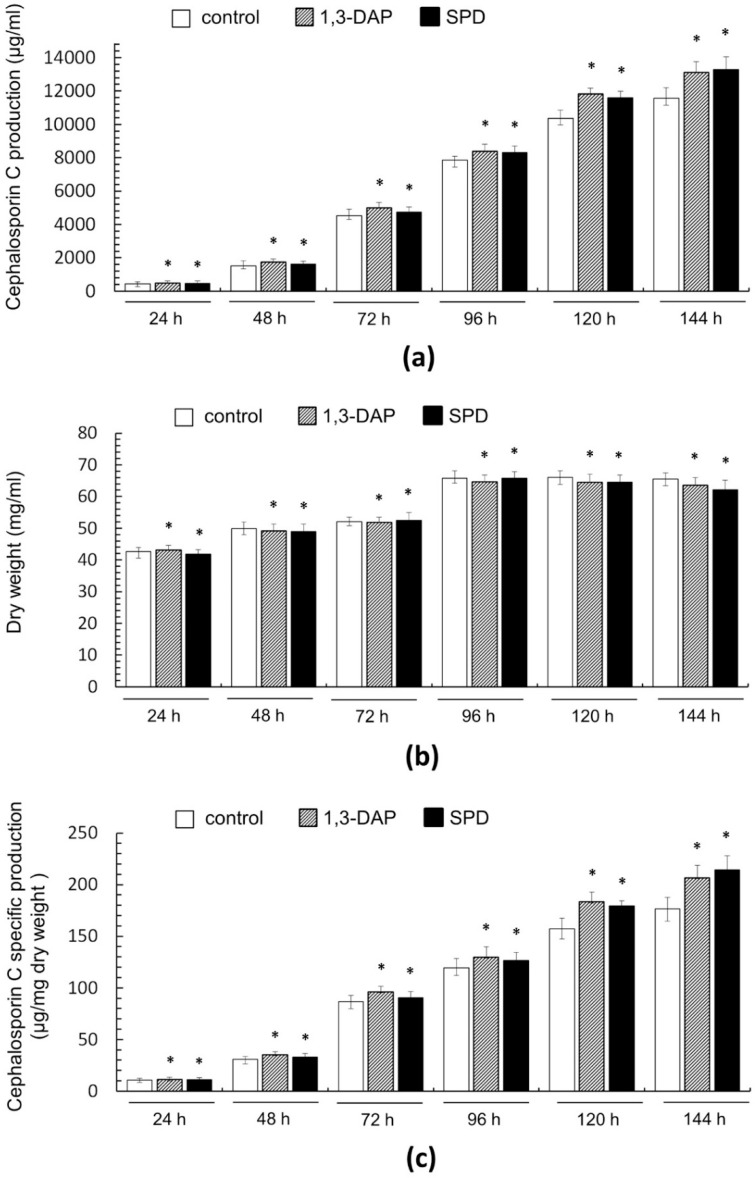
Effect of the addition of 5 mM 1,3-DAP or 5 mM spermidine (SPD) on cephalosporin C (CPC) production and growth of *A. chrysogenum* HY strain during fermentation on complex (CP) medium: (**a**) CPC production; (**b**) dry weight; (**c**) CPC specific production. Samples were taken at 24 h, 48 h, 72 h, 96 h, 120 h, and 144 h. Data are means ± SD, *n* = 3. Statistical significance, * *p* ≤ 0.05, as compared with the control (strain, cultivated on medium without PAs additions).

Determination of the most effective concentration and a suitable period for introducing exogenous PAs allowed, at the next stage of work, to study the dynamics of the stimulating effect on the production of CPC in *A. chrysogenum* HY (Figure 5a).

Samples were analyzed every 24 h from the beginning of cultivation on the CP medium until the end of the process after 144 h (corresponds to 13–19 days of work with the strain, according to Figure 4). We also studied biomass accumulation during fermentation (Figure 5b), which allowed us to determine the specific yield of CPC per unit dry weight (Figure 5c).

**Figure 6:**
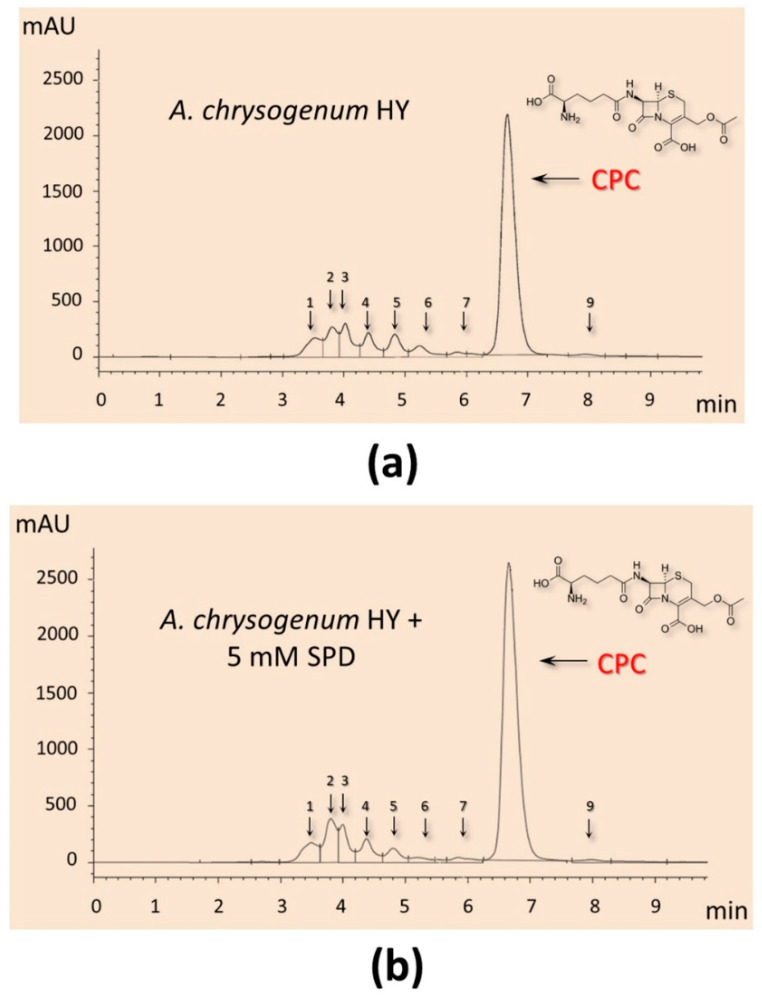
HPLC analysis of beta-lactam production of the *A*. *chrysogenum* high-yielding (HY) strain after 144 h of cultivation on complex (CP) medium: (**a**) cultivation without additives (control); (**b**) with 5 mM spermidine (SPD) added at the starting point of cultivation.

It turned out that the addition of PAs did not have a significant effect on the production of CPC on the first day of fermentation (Figure 5c). Further, within 48–96 h, a small but distinct stimulating effect was observed. Cultivation in the presence of 5 mM 1,3-DAP increased CPC production by 7–14%; the addition of 5 mM SPD increased CPC production by 5–6%. The stimulating effect of adding 5 mM PAs was manifested in the late stage of fermentation. After 120–144 h of cultivation with 5 mM 1,3-DAP, the production of CPC was 13–14% higher than in the control. The addition of SPD led to an increase in the yield of SPD by 11–12% after 120 h and up to 15% after 144 h (Figure 6). In absolute terms, after 144 h, the HY strain on the SP medium without additive produced ~11,500 mg/L of CPC; the SP medium supplemented with 5 mM SPD produced ~13,300 mg/L of CPC (Figure 5c and Figure 6). At the same time, there was no increase in the relative level of beta-lactam biosynthesis byproducts such as deacetoxycephalosporin C (DAOC) or deacetylcephalosporin C (DAC), which was observed previously after some manipulations with this strain [2,15] (Figure 6).

The active growth of the biomass of the *A. chrysogenum* HY strain during cultivation on the CP medium without additives occurred for up to 96 h; then, a stationary phase began. The amount of biomass practically did not change until the end of fermentation. (Figure 5b). The addition of PAs had no significant effect on biomass production until the final fermentation period, when a slight decrease was observed, up to 3%, when cultured with 5 mM 1,3-DAP and up to 5% when cultured with 5 mM SPD.

A slight decrease in the amount of biomass accompanying an increase in CPC production at the final stage of fermentation with PAs led to an even more significant increase in the specific CPC production, expressed in μg/mg dry weight, compared to the control (Figure 5c). The addition of 5 mM DAP increased the specific production of CPC after 120–144 h by 16–17%. The effect of adding 5 mM SPD was even greater; after 120 h, the specific production of CPC increased by 14%; after 144 h, the increase reached 20–21%.

### 2.3. Analysis of the CPC Production and Expression Level of the Corresponding Biosynthetic Genes in the A. chrysogenum HY Strain after the Addition of Exogenous PAs during Fermentation

The study of the effect of PAs on the level of CPC biosynthesis during fermentation of *A. chrysogenum* HY strain made it possible to choose the optimal time of their introduction to increase the production of this SM. Since the maximum effect from the addition of PAs was found at the final stage of fermentation, to determine the molecular basis of such an increase in CPC production, the expression level of the “early” and “late” beta-lactam genes was measured, starting from 1 h after the transfer of the culture from the DP medium to the CP medium until the end of fermentation after 144 h. Samples were collected every 24 h.

We studied the expression of biosynthetic genes of the “early” beta-lactams BGC: (i) gene pcbAB for ACV (δ-[l-α-Aminoadipyl]-l-Cysteinyl-d-Valine) synthetase (EC: 6.3.2.26), the central enzyme in the biosynthesis of beta-lactams, which creates in ACV tripeptide as NRPS (nonribosomal peptide synthetase); (ii) gene pcbC for isopenicillin N-synthase (EC: 1.21.3.1), oxygenase, which synthesizes penicillin N (IPN) from the ACV tripeptide; (iii) gene cefD1 for isopenicillin N-CoA synthetase (EC: 5.1.1.17), IPN epimerase component 1, which activates IPN by the acyl-CoA synthase; (iv) gene cefD2 for isopenicillin N-CoA epimerase (EC: 5.1.1.17), IPN epimerase component 2, which epimerizes IPN-CoA to penicillin N (PenN). We also studied the expression of biosynthetic genes of the “late” beta-lactams BGC: (v) gene cefEF for deacetoxycephalosporin C synthetase (penN expandase) (EC 1.14.20.1)/deacetoxycephalosporin C hydroxylase (1.14.11.26), which sequentially catalyzes two oxygenase reactions for the conversion of PenN to DAOC and then to DAC; (vi) gene cefG for deacetylcephalosporin-C acetyltransferase (EC 2.3.1.175), which transfers the acetyl residue from acetyl coenzyme A to the DAC to produce CPC.

**Figure 7:**
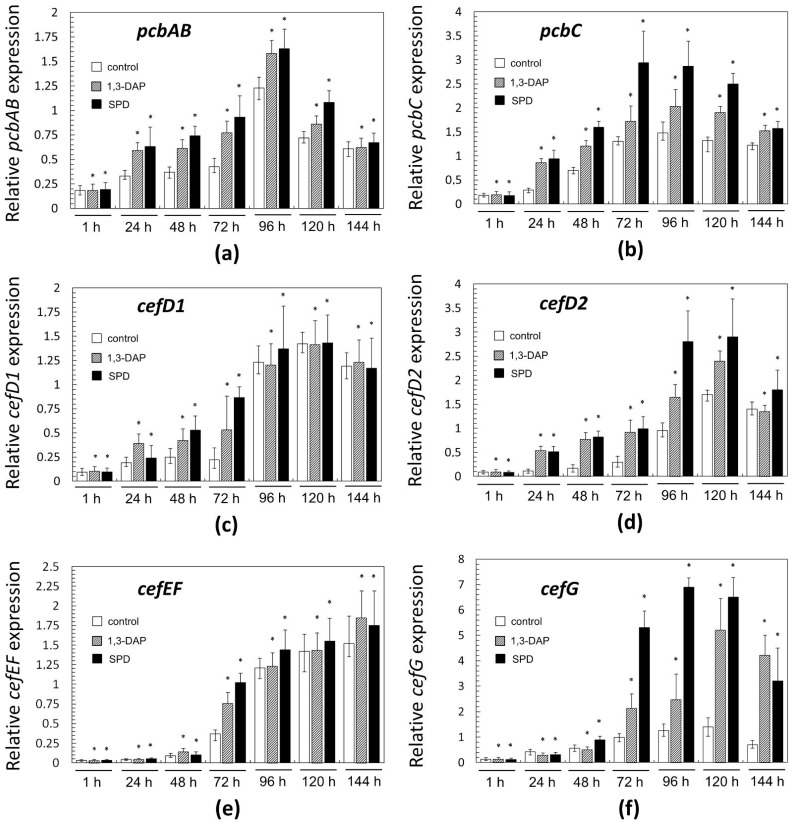
Expression dynamics of: (**a**) *pcbAB*; (**b**) *pcbC*; (**c**) *cefD1*; (**d**) *cefD2*; (**e**) *cefEF*; (**f**) *cefG* genes in *A. chrysogenum* HY strain after the addition 5 mM 1,3-DAP or 5 mM spermidine (SPD). After 1, 24, 48, 72, 96, 120, and 144 h of fermentation on complex (CP) medium. Data are means ± SD, *n* = 3. Statistical significance, * *p* ≤ 0.05, as compared with the control (strain, cultivated on medium without PAs additions).

A total of 1 h after the transfer of the culture from the DP medium to the CP medium with or without PAs no differences in the expression of genes for the biosynthesis of beta-lactams were observed (Figure 7).

Then, the dynamics of expression for “early” and “late” genes of beta-lactam biosynthesis differed. In control samples (collected from the CP medium without additives), the level of mRNA expressed from the “early” genes smoothly increased in the range of 24–96 h, for *pcbAB* and *pcbC*, or in the range of 24–120 h, for *cefD1* and *cefD2*; after a period of growth in gene expression, there was a slight drop in this level in all cases (Figure 7a–d). In the control for “late” genes, the expression level changed slightly, up to the middle of fermentation, 72 h; and then a rapid increase in the level of expression began at 72–120 h. By the end of the fermentation, the expression level either increased slightly for *cefEF* or decreased for *cefG*.

The addition of PAs in most cases either did not change the level of expression of both “early” and “late” genes (more often it manifested itself in the early stages of fermentation) or led to their upregulation (more often it manifested itself in the middle and end of fermentation). If upregulation for a certain gene occurred at a certain time point, then in most cases, it was caused by both polyamines; the upregulation with the addition of spermidine was generally slightly higher; it could reach 6–8-fold. In only a few cases, 1,3-DAP upregulated the studied genes more strongly than spermidine, for example, *cefD1* after 24 h or *cefG* after 144 h. Among the biosynthetic genes of beta-lactams, *cefG* was most strongly upregulated in the period 72–144 h; the addition of 1,3-DAP upregulated *cefG* 2–4 times; the addition of spermidine upregulated *cefG* 3–8 times, compared with the control.

## 3. Discussion

SMs in filamentous fungi are synthesized in the idiophase, which replaces the first phase of development, the tropophase [48]. The addition of PAs on an agar medium stimulates, in the wild-type strain, the appearance of the characteristic yellow-cream coloration associated with the biosynthesis of the SM of sorbicillin a few days earlier (Figure 2). This effect of PAs on accelerating the functioning of the secondary metabolic pathway may be more universal. It has previously been shown that the addition of 5 mM 1,3-DAP or 5 mM spermidine enhances the yellow-green pigment coloration of *Penicillium chrysogenum* 54–1255 by increasing the production of the yellow compound component chrysogenin [27]. Additionally, it was shown that PAs could stimulate an earlier production of the target SM in improved fungi strain during submerged fermentation. Thus, the addition of 5 mM 1,3-DAP or 5 mM SPS made it possible to obtain lovastatin during the fermentation of high-yielding *A. terreus* 3 days earlier than in the control [28]. Targeted knockout of the *sorA* or *sorB* genes (encoding the central enzymes, polyketide synthases of sorbicilinoid BGC) in the *A. chrysogenum* wild-type strain results in a white phenotype, exactly the same as in *A. chrysogenum* HY [43]. It can be assumed that in *A. chrysogenum* HY, the biosynthesis of sorbicillin was disrupted; therefore, the yellow-cream color does not develop either during the idiophase period or under the influence of polyamines. This may be due to screening after random mutagenesis when mutants with disruption of the biosynthesis of alternative SM are also selected [49]. The inability of the improved strains to synthesize some alternative SM was shown at the molecular level and may be due to the release of additional resources for targeted biosynthesis [49]. Disruption of the biosynthesis of polyketide sorbicillin could lead to an additional increase in the production of cephalosporin C in *A. chrysogenum* HY.

The addition of PAs also showed the stimulation of colonial germination, quantified by an increase in CFU/mL (Figure 3e). This is due to the fact that the improved producer is an extremely weakened strain, and even on a complete medium, not all colonies germinate. Thus, OD_600_ = 0.5 were ~2.7 × 10^7^ CFU/mL for the *A. chrysogenum* WT strain and 1.6 × 10^6^ CFU/mL for the *A. chrysogenum* HY strain. Industrial strains with overproduction of SMs, obtained using classical strain improvement (CSI) programs, often contain unwanted side mutations and “bottlenecks” that negatively affect the fitness of the strain, resistance, productivity, and adaptation to harsh fermentation conditions [2,50,51]. It is possible that the introduction of exogenous PAs could complement or correct some of these negative effects. A similar stimulating effect on the germination of colonies after adding 5 mM PAs to the agar culture medium was shown for a high-yielding *Aspergillus terreus* strain, a producer of lovastatin [28].

The addition of PAs on the agar medium also increased the viability of the HY strain in terms of colony size (Figure 3e). The heterogeneity in the size of the colonies was removed; small and medium colonies reached the size of large colonies for this strain. In addition, extremely weakened colonies germinated, which did not appear on the medium without polyamines (and they were also large in size). Due to this, the total number of colonies increases by about 3–5 times with the addition of 5 mM PAs. The addition of spermidine further increased the colony size. Colonies that actively produce CPC are smaller than large nesting colonies that lose high activity [11]. In this regard, it was important to determine whether the increase in the viability of the HY strain upon the introduction of PAs is accompanied by a loss of high CPC activity. This was revealed in submerged fermentation, where we were able to find the conditions under which the addition of PA led to an increase in the production of the CPC of the HY strain (Figure 4 and Figure 5).

The observed effect of the increase in the size and number of HY strain colonies upon the addition of PAs could not be scaled in the case of submerged fermentation. The addition of PAs did not lead to an increase in biomass compared to the control (Figure 5b). In addition, a slight decline, by 3–5%, was observed in biomass production when cultivated with PAs in the later stages of fermentation. Cultivation after 144 h, both with added PA and in the control, resulted in a sharp drop in biomass and the content of CPC. In another study, at the end of fermentation with PAs of the high-yielding strain *A. terreus*, the decrease in biomass was more significant and reached 10–15% [28]. Additionally, a decrease in biomass occurred at the end of fermentation with PAs of the improved *P. chrysogenum* Wis 54–1255, by 3–5% [27]. Possibly, the concentration of PAs that stimulate SM production in improved strains is toxic at the last stages of fermentation or leads to earlier aging.

In our work, for the *A. chrysogenum* HY strain, the conditions were optimized where the final yield of CPC was increased by 10–15% due to the introduction of PAs. Earlier, when working with *A. chrysogenum* HY, we could not obtain a CPC yield of more than 12,000 mg/L during fermentation in flasks. With the addition of 1,3-DAP, the yield of CPC increased to 13,100 mg/L; with the addition of SPD, it increased to 13,300 mg/L (Figure 5a). This is an important result that can be realized in practice. Earlier, for an improved producer of *A. terreus*, the addition of exogenous PAs increased the production of lovastatin by 20–45%, but this increase was on day 8 (out of 11) of fermentation [28]. Further, the production of lovastatin decreased; the practical significance, in this case, was associated with obtaining the same amount of lovastatin as in the control, but 3 days faster, thanks to the addition of PAs.

It is also important that the addition of PAs has a balanced effect on beta-lactam metabolism in *A. chrysogenum* HY. The total increase in the admixture products of beta-lactam metabolism does not exceed the increase in the production of CPC (Figure 6). Due to this, the percentage of admixtures after fermentation of *A. chrysogenum* HY with PAs remains at the control level, 10–15%.

This shows that PAs effectively influence the entire biosynthetic pathway of beta-lactams in *A. chrysogenum* HY; no limiting stage arises, leading to an increased content of the intermediate biosynthesis product. Thus, in the previous work with this strain, the expression of an additional copy of the cefT gene (encoding the beta-lactam transporter) led to an increase in intermediate biosynthesis products (IPN, DAOC, and DAC) and a decrease in the yield of CPC [15]. In another study, an increase in the activity of H^+^-ATPase plasma membrane (PMA) in recombinant *A. chrysogenum* HY strains correlated with an increase in DAC and a decrease in CPC production as a result of ATP deficiency [2]. For the last stage of beta-lactam biosynthesis, cytoplasmic acetyl-coenzyme A is required as a substrate; its synthesis consumes ATP. PMA is the main enzyme that consumes cell ATP; in recombinant clones with increased PMA activity, the ATP content sharply decreases, disrupting high-energy beta-lactam biosynthesis, especially at the last stage [2]. The fact that an increase in CPC production with the addition of PAs does not lead to an increase in the percentage of impurities is important, from a technological point of view, when isolating and purifying the substance (Figure 6).

Earlier, we showed that in an *A. chrysogenum* HY strain, the “early” and “late” genes of beta-lactam BGCs are upregulated 5–300 times compared to the *A. chrysogenum* WT strain [13]. In the current study, it was shown that the introduction of PAs leads to additional upregulation of all six genes for the biosynthesis of beta-lactams (*pcbAB*, *pcbC*, *cefD1*, *cefD2*, *cefEF*, and *cefG*) (Figure 7). Since we previously studied the expression of these genes only after 0 (at the time of inoculum from DP to CP medium), 48, and 120 h, the study of additional time points allowed us to more clearly trace the dynamics of their expression [13]. It was possible to trace exactly when the action of polyamines at the molecular level is turned on. For “early” genes, this impact was in the period of 24–120 h (Figure 7a–d), for “late” genes—in the period 72–144 h (Figure 7e,f). Detection of *cefG* upregulation is especially significant since the final stage of the CPC biosynthetic pathway is rate-limiting and estimated as a “bottleneck” for CPC biosynthesis [2]. Several improved *A. chrysogenum* strains produce significant amounts of DAC byproduct due to insufficient activity of the CefG enzyme, which significantly reduces the yield of the target metabolite, CPC [52]. The fact that the increase in the production of CPC under the influence of PAs in the HY strain is not accompanied by a significant increase in byproducts, especially DAC, can be partly explained by the increased expression of *cefG* under the influence of PAs (Figure 6 and Figure 7f).

## 4. Materials and Methods

### 4.1. Materials

1,3-diaminopropane (1,3-DAP) and spermidine (Spd) were obtained from MP Biomedicals.

### 4.2. Strains of Microorganisms

*A. chrysogenum* ATCC 11550 (WT, wild type Brotzu isolate, [53]) and *A. chrysogenum* RNCM 408D (HY, high-yielding CPC producer, derived from the WT, [11]) were used in this work.

### 4.3. Cultivation of A. chrysogenum Strains on Agarized Media with PAs

*A. chrysogenum* strains were cultivated on agarized complex (CPA) medium (40 g/L maltose, 10 g/L peptone, 20 g/L malt extract, 25 g/L agar, pH 7.0–7.4), or agarized Czapek–Dox (CDA) medium (30 g/L sucrose, 2 g/L NaNO_3_, 1 g/L K_2_HPO_4_, 0.5 g/L MgSO_4_·7H_2_O, 0.5 g/L KCl, 0.01 g/L FeSO_4_·7H_2_O, 25 g/L agar, pH 7.0–7.4), or agarized LPE medium (10 g/L glucose, 20 g/L yeast extract, 15 g/L NaCl, 10 g/L CaCl_2_, 25 g/L agar, pH 6.8). CPA medium was supplemented with 1,3-DAP or SPD in the concentration range 0.1–10 mM or used without additions (control).

To determine the effect of PAs on the growth and morphology of *A. chrysogenum* colonies, the serial dilution method was used. *A. chrysogenum* cells were collected from CPA slants and diluted with 0.9% NaCl up to OD_600_ = 0.5 (basic concentration), followed by 6–7 serial tenfold dilutions with the same solvent. Then, 50 μL of cell suspension was inoculated onto Petri dishes (with CPA medium prepared with or without the addition of 0.1–10 mM 1,3-DAP or SPD), incubated at 28 °C for 10–15 days for *A. chrysogenum* WT cells or 18–25 days for *A. chrysogenum* HY. Colonies on a medium with PAs were compared with colonies in control, in dilutions with 5–500 colonies. The effect of the PAs’ addition on the number of germinating colonies was evaluated as ratio of CFU/mL (colony forming units) of cell counts after incubation with PAs, compared with control counts (on CPA media without any additions), i.e., normalized to control. For reference, the average control counts were approximately 2.7 × 10^7^ CFU/mL for *A. chrysogenum* WT strain and 1.6 × 10^6^ CFU/mL for *A. chrysogenum* HY strain.

The effect of PAs on the colony size was calculated as the ratio of the average diameter of all colonies after inoculation on CPA with PAs compared to control’s average diameter of colonies (on CPA media without any additions that were grown from the inoculum of the same dilution). The counting of the number and size of colonies was carried out after 5 days of incubation for the WT strain and after 12 days of incubation for the HY strain. Data represent triplicates from four separate experiments, with the mean and SEM displayed.

### 4.4. Submerged Fermentation of A. chrysogenum HY Strain with Exogenous PAs

*A. chrysogenum* HY strain was routinely cultured on CPA slants. To prepare the strain for antibiotic production, it was inoculated from CPA on LPE slants, incubated 10 days at 28 °C; the whole content collected from agar with 5 mL 0.9% NaCl, transferred to 25 mL of the defined (DP) medium (28 g/L yeast extract, 28 g/L malt extract, 10 g/L peptone, 4 g/L chalk, 20 g/L soybean oil, pH 7.2) in 250 mL Erlenmeyer flasks, and incubated on a rotary shaker at 220–240 rpm at 28 °C. After 48 h of growth, 20 mL of culture was inoculated in 35 mL of complex (CP) medium (105 g/L corn extract, 60 g/L corn dextrin, 20 g/L corn starch, 3 g/L KH_2_PO_4_, 5 g/L glucose, 3.5 g/L MgSO_4_, 14 g/L (NH_4_)_2_SO_4_, 11 g/L chalk, 20 g/L soybean oil; supplemented with microelements: 18 mg/L CuSO_4_·5H_2_O, 150 mg/L ZnSO_4_·7H_2_O, 30 mg/L MnSO_4_·7H_2_O, 70 mg/L FeSO_4_·7H_2_O, pH 6.2–6.4). Fermentation was performed in 750 mL Erlenmeyer flasks for 144 h (240 rpm) at 28 °C for the first 24 h and at 24 °C for the rest of the process. A total of 0.5–5 mM 1,3-DAP or SPD was added at various stages of the preparation of HY strain for fermentation and the fermentation itself: (i) preliminary cultivation on LPE slants; (ii) two consecutive passages on slant agar, each time with the addition of PAs; (iii) at the time of inoculum from LPE slants to DP medium; (iv) at the time of inoculum from DP medium to CP medium; (v) after 24 h of cultivation on CP medium; (vi) after 72 h of cultivation on CP medium.

### 4.5. Determination of Dry Biomass

Aliquots (2 mL), which included medium and cells, were taken after 24 h, 48 h, 72 h, 96 h, 120 h, and 144 h of growth, centrifuged in a 15 mL falcon at 4800× *g*, washed three times with 10 volumes of H_2_O and placed in a thermostat at 80 °C. Drying was carried out for 48–72 h until a constant weight was established. Dry biomass was determined by the difference between the weight of dried cells and empty falcon. Data represent triplicates from four separate experiments, with the mean and SEM displayed.

### 4.6. HPLC Analysis of Beta-Lactams

To determine the yield of cephalosporin C and byproducts of the biosynthesis of beta-lactams, aliquots of the culture fluid were taken after 24 h, 48 h, 72 h, 96 h, 120 h, and 144 h of growth. The concentration beta-lactams in the culture broth was determined in the mobile phase CTAB/acetonitrile/phosphoric acid/water on a chromatographic column YMC-Pack ODS-A (YMC CO., Kyoto, Japan) with a particle diameter of 5 μm at a flow rate of the mobile phase of 1.0 mL/min, and a detection wavelength of 254 nm. Data represent triplicates from four separate experiments, with the mean and SEM displayed.

### 4.7. Preparation of Total RNA and cDNA Synthesis and qPCR Analysis

Cell samples for total RNA extraction were taken at 1 h, 24 h, 48 h, 72 h, 96 h, 120 h, and 144 h of growth, filtered, washed with PBS, lyophilized, and stored at −80 °C. The total RNA preparation and cDNA synthesis were carried out as described previously [13,15]. qPCR reactions were performed with previously developed pairs of primers for analysis of gene expression of CPC biosynthesis (*pcbAB*, *pcbC*, *cefD1*, *cefD2*, *cefEF*, and *cefG*) (Table 1) [2,13]. Reactions and processing of the results were carried out in accordance with the protocol [13]. To normalize the data of expression levels, we used previously designed pair of primers for the housekeeping γ-actin gene [15]. Data represent triplicates from four separate experiments, with the mean and SEM displayed.

### 4.8. Statistical Analysis

The experimental data were expressed as mean value ± standard error of mean (SEM) calculated from three parallel experiments. The statistical analysis was performed by one-way analysis of variance (ANOVA) using Microsoft Excel. Differences described by *p* ≤ 0.05 were considered significant.

## 5. Conclusions

In our work, we showed that the introduction of exogenous polyamines could additionally increase the production of cephalosporin C in a high-yielding *Acremonium chrysogenum* strain, by 10–15%. This was accompanied by an upregulation of both “early” and “late” genes from the biosynthetic clusters of beta-lactams, especially *cefG*, which encodes a key enzyme of the final biosynthesis stage that converts deacetylcephalosporin C to cephalosporin C. Since it was previously shown that exogenous polyamines could increase the production of other improved fungi producers, in particular, the production of penicillin G and lovastatin, our results may reflect a certain general trend that can be implemented in the cultivation of industrial fungi strains.

## Figures and Tables

**Table 1:** Primers used for RT-PCR analysis.

Primer	Gene	Product, Function	Oligonucleotide (Sequence 5 → 3)	Source Sequence
actq1	*act1*	γ-actin, a major component of the cytoskeleton	CCGGTTTCGCCGGTGATGATGCT	JN836733.1 [15]
actq2			TGCTCAATGGGGTAGCGCAG	
pcbABq3	*pcbAB*	δ-(l-α-aminoadipyl)-l-systeinyl-d-valine synthetase	AGGCATCGTCAGGTTGGCCG	E05192.1 [13]
pcbABq4			CCGGAGGGGCCATACCACAT	
pcbCq1	*pcbC*	isopenicillin N-synthase	CTAGGTCGCGACGAGGACTTCT	M33522.1 [13]
pcbCq2			CACGTCGGACTGGTACAACACC	
cefD1q1	*cefD1*	isopenicillin N-CoA synthetase	CCCCGGTGAGGAAGATGCGT	AJ507632.2 [13]
cefD1q2			TCGATCTCCGCCTTGGACGC	
cefD2q1	*cefD2*	isopenicillin N-CoA epimerase	ACAGGATGGAGAGGAGCACCTTG	
cefD2q2			TCGTAGAGCTCGCGGGGCTA	
cefEFq3	*cefEF*	deacetoxycephalosporin C synthetase/hydroxylase	GTCGAGTGCGATCCCCTCCT	AJ404737.1 [2]
cefEFq4			CGAATTCTCCGTCCACCTCG	
cefGq3	*cefG*	deacetylcephalosporin-C acetyltransferase	ATCTCAGTCTCCGAAGCGTCCTGG	M91649.1 [2]
cefGq4			CGAGGATTTGTGACCGACATAAGTGG	

## Data Availability

The data presented in this study is contained within the article.

## Sample Availability

Samples of the compounds are not available from the authors.
